# Addressing the shortage of personal protective equipment during the COVID-19 pandemic in India-A public health perspective

**DOI:** 10.3934/publichealth.2020019

**Published:** 2020-04-15

**Authors:** Sudip Bhattacharya, Md Mahbub Hossain, Amarjeet Singh

**Affiliations:** 1 Department of Community Medicine, Himalayan Institute of Medical Sciences, Dehradun, India; 2 Department of Health Promotion and Community Health Sciences, School of Public Health, Texas A & M University, Texas, USA; 3 Department of Community Medicine & School of Public Health, PGIMER, Chandigarh, India

**Keywords:** pandemic, personal protective gears, corona virus, COVID-19

Whenever any pandemic accelerates, per se the coronavirus disease 2019 (COVID-19), it is commonly observed that health care systems face tremendous workload in terms of infectious patients seeking testing and care. During such public health emergencies, personal protective equipment (PPE) like-gloves, surgical face masks, air-purifying respirators, ventilators, goggles, face shields, N95 respirators, and gowns are essential in preventing the spread of infection among the patients and health care workers (HCWs). As in this critical phase, a shortage of all of these PPE is about to develop or has already developed in high demand areas like triage, isolation wards etc. Previously, PPE was commonly used in the hospital environment, is now a scarce and precious commodity in many locations when it is needed most to care for highly infectious patients [1].

It is even more difficult to get PPE when common people get started to use/stock PPE in fear of infectious disease contamination without following national guidelines, which is an added insult to the injury of health system. During a pandemic, whether it is striking in developing or developed countries, an increase in the supply of PPE in response to this new demand will require a large increase in the production and distribution of those equipment. However, most of the time it is not possible to manufacture bulk PPE as it requires time, infrastructure, and other resources. In a pandemic situation, a hospital is unlikely to share their PPE and other protective resources to other hospitals or medical institutions as they may require it too for ensuring their own safety [1]. Moreover, rapid coordination of such resources in the state or national level can be useful so that equipment are not being used can be mobilized with other institutions experiencing scarcity. Such approaches may foster collaborative efforts against COVID-19 ensuring efficient use of resources at the systems level. Nonetheless, it is only possible to address COVID-19 if we can flatten the epidemic curve by classical intervention measures like lockdown and social distancing processes, which may give lead time to many health care systems to arrange further management of the outbreak. But during exponential phase of pandemic as rapid increase in COVID-19 patients it is very challenging to provide adequate PPEs to the health workers of any country. To solve this problem, i.e., to optimize the use of face masks during the pandemic, the Centers for Disease Control and Prevention (CDC) identifies 3 levels of operational status: conventional, contingency, and crisis [1]. During normal times, face masks are used in conventional ways to protect HCWs from splashes and sprays. When health care systems become stressed and enter the contingency mode, CDC recommends conserving resources by selectively cancelling nonemergency procedures, cancelling outpatient encounters which might require face masks/PPEs.

When face masks are unavailable, the CDC recommends use of face shields without masks, taking clinicians at high risk for COVID-19 complications out of clinical service, staffing services with convalescent HCWs presumably immune to SARS-CoV-2 (severe acute respiratory syndrome coronavirus 2), and use of homemade/handmade masks, perhaps from bandanas or scarves if necessary [2]. Many communities in the India and globally are rapidly entering PPE crisis mode. Recently news are circulating about the unconventional solutions for PPE at local hospitals, such as plastic garbage bags for gowns and plastic water bottle cut outs for eye protection [3]. Shortage of sanitizer can be solved by using handmade sanitizer having 90% concentration of alcohol, this type of ideas/news/decisions are facing many continued criticism from medical fraternity as they are perceiving as mockery/knee jerk response. Plans for resupply through the repurposing of existing industrial capacity are welcome but seem unlikely to solve the shortage quickly enough as supply chains become affected in the pandemic [4].

The task force to combat COVID-19 was created to solve precisely this problem, but its inventory is not transparent and news reports suggest its supplies are being distributed unevenly or are insufficient to meet demand [5]. HCWs need supplies and solutions for these shortages now, and for that reason, the Journal of American Medical Association (JAMA) issued a call for ideas for how to address the impending PPE shortage [6]. There were many proposals (Table 1).

**Table 1.** Methods of PPE conservation and management.

Import-Purchase PPE	Use of smart technology-drones, telemedicine, etc
Reuse-by sterilization	Employ healthy workers
Reduce non-essential services	Use government solutions
Reduce patient contact	Use innovative solutions
Alter staffing	Stratify use by risk profiling
Rely on local solutions	Manage supply

One endeavour is Project N95, where demands are identified and N95 masked to be supplied on that area only [6]. Sterilization of used PPE with agents ranging from ethylene oxide, UV or gamma irradiation, ozone, and alcohol was identified as common proposal. There were also novel proposals such as mask-fiber impregnation with copper or sodium chloride, these ideas are not unscientific they were field tested after prior viral epidemics to determine the feasibility of sterilizing PPE [7]. Although scientists acknowledged that the uncertainty about the effects of these sterilizing agents on the structural integrity of PPE, and there is some evidence the fibers in masks and respirators that filter viral particles can degrade and lose their efficacy with PPE reprocessing [7].

Some of the other idea was to reduce patient contact so most of the private clinics remains closed and most of the clinicians doing teleconsultations. Alter staffing is also considered as important step, health department of India gave directives to the medical colleges that the all health care workers will work on a rotation basis for minimizing the contact risk [8].

Home delivery of online groceries are another option. In India, a company named “*Big-Bazaar*” is already providing online groceries to the peoples who are confined in their homes due to lockdown [9]. However, such technology-based services are contingent on the availability and accessibility of those services in different countries. In India and other low and middle-income countries, innovative technological interventions should be devised and deployed to ensure timely and efficient distribution of goods and services. Such socioeconomic approaches may not only reduce the risks of COVID-19 transmission but also ensure daily necessities of the citizens are met adequately.

Other measures are like appointing the healthy staffs to the service area and the staffs who have medical conditions are exempted from service delivery.

Other than that, using government services like relaxing importing rules, use of police forces, converting railway coaches as isolations are also important and innovative steps.

Legislative steps like mandatory social distancing, curfew, can help the crisis period by flattening the epidemic curve [10].

These are the short-term conventional solutions. Here we propose few more which is out of the box thinking like-production of sanitizer at mass scale by the alcohol industry during COVID-19 crisis period, in India is happening right now [11]. Similarly, in India, the textile industry and hardware industry is producing bulk masks, gowns, caps, protective shields etc instead of producing clothes [12]. Moreover, the automobile industry can make ventilators instead of producing vehicles at this critical period. In India, the Mahindra group came out with a prototype ventilator and soon they will start producing [13].

Besides this, global evidence on managing the shortage of PPE can be useful to inform future strategies. For example, Taiwan experienced a critical shortage at the beginning of the COVID-19 crisis. Implemented this issue was mitigated by several strategies including rapid production and distribution of PPE to prioritized centres resulting in a declined shortage of PPE. These strategies used a 3-tier personal protective equipment (PPE) stockpiling framework that could maintain a minimum stockpile for the surge demand of PPE in the early stage of a pandemic [14]. Some of these strategies include export prohibition, rationing, and increase production through either mandates or voluntary productions [15]. We believe many lessons can be learnt from countries like these. These countries provide real-time examples that can be copied by others with similar healthcare systems.

Such local and global innovations should be evaluated and adopted ensuring patient compliance during COVID-19 to improve health outcomes.

In our opinion smart questions need smart answers, in the era of emerging and re-emerging disease outbreaks like COVID-19, besides the conventional approach we must think differently and implement the success stories of similar countries in India. While health systems in most of the countries are struggling to fight COVID-19, the operational challenges including safety of the health workforce and prevention of transmission is much higher in resource-constrained contexts. It is essential to prioritize these health issues and adopt best practices to ensure the availability, accessibility, and utility of PPE and other resources in an efficient way. Multilevel policy interventions with user-level quality assurance may help in mitigating those issues. Perhaps, more importantly, we have to extend our support to each other, act together for our survival, without blaming each other.
